# Supplementary material for: Structural resolution of inorganic nanotubes with complex stoichiometry
Source: Nat Commun. 2018 May 23;9:2033. doi: 10.1038/s41467-018-04360-z (PMC5964105; doi:10.1038/s41467-018-04360-z)
Supplement: Supplementary file 1 — Supplementary Information [file 41467_2018_4360_MOESM1_ESM.pdf]

Supplementary Information for

Structural resolution of inorganic nanotubes with complex stoichiometry

Geoffrey Monet[†], *Mohamed S. Amara*[†], *Stéphan Rouzière*[†], *Erwan Paineau*[†], *Ziwei Chai*[§,○], *Joshua D. Elliott*[‡,a], *Emiliano Poli*[‡,b], *Li-Min Liu*[c,§], *Gilberto Teobaldi*[§,‡,*] *and Pascale Launois*[†,*]

[†]Laboratoire de Physique des Solides, UMR CNRS 8502, Université Paris Sud, Université Paris Saclay, 91405 Orsay Cedex, France, [§]Beijing Computational Science Research Centre, 100193 Beijing, China, [‡]Stephenson Institute for Renewable Energy and Department of Chemistry, The University of Liverpool, L69 3BX Liverpool, United Kingdom, [a]Present address: Dipartimento di Fisica e Astronomia "Galileo Galilei", Università degli Studi di Padova, I-35131 Padova, Italy & CNR-IOM DEMOCRITOS c/o SISSA, 34136 Trieste, Italy, [b]Present address: The Abdus Salam International Centre for Theoretical Physics, 34151 Trieste, Italy, [c]School of Physics, Beihang University, 100191 Beijing, China, [○]First Density Functional Theory author.

*Corresponding Authors

Supplementary Note 1. Geometrical energy E_{geo}

The energy E_{geo} is the sum of harmonic energies of bonds and angles between bonds within the unit cell. A diamond-shaped unit cell defined by the vectors **a** and **b**, strictly equivalent to the one in Figure 1 in the manuscript, is drawn in Supplementary Figure 1. Alternatively, we introduce γ the angle between **a** and **b** and a their norm. A quadratic term $E_{\text{AB}} = \frac{k_{\text{AB}}^0}{2}\left(d_{\text{AB}} - d_{\text{AB}}^0\right)^2$ is associated to the bond between atoms A and B (A and B can also be 'virtual' atoms representing OH and CH_3 entities, see the article), d_{AB} being the bond length and d_{AB}^0 (respectively, k_{AB}^0) a reference bond distance (resp. spring constant) gathered from the literature (Supplementary Table 1). Similarly, a harmonic term $E_{\widehat{\text{ABC}}} = \frac{k_{\widehat{\text{ABC}}}^0}{2}\left(\theta_{\widehat{\text{ABC}}} - \theta_{\widehat{\text{ABC}}}^0\right)^2$ is associated to the angle $\widehat{\text{ABC}}$ between bonds AB and BC. Thus, the total harmonic energy writes:

$$E_{\text{geo}}\left(\{\boldsymbol{r}_k\}_{N_{\text{at}}}\right) = \sum_{\text{AB } bounds} \frac{k_{\text{AB}}^0}{2}\left(d_{\text{AB}} - d_{\text{AB}}^0\right)^2 + \sum_{\widehat{\text{ABC}}\ angles} \frac{k_{\widehat{\text{ABC}}}^0}{2}\left(\theta_{\widehat{\text{ABC}}} - \theta_{\widehat{\text{ABC}}}^0\right)^2 \#(1)$$

$\boldsymbol{r}_k$ being the position of atom k in the unit cell, $d_{\text{AB}} = \|\boldsymbol{r}_{\text{B}} - \boldsymbol{r}_{\text{A}}\|$ the distance between atoms A and B, $\theta_{\widehat{\text{ABC}}}$ the angle between $\boldsymbol{r}_{\text{A}} - \boldsymbol{r}_{\text{B}}$ and $\boldsymbol{r}_{\text{C}} - \boldsymbol{r}_{\text{B}}$. $N_{\text{at}} = 10$ is the number of atoms in the unit cell as displayed in the Supplementary Figure 1 below.

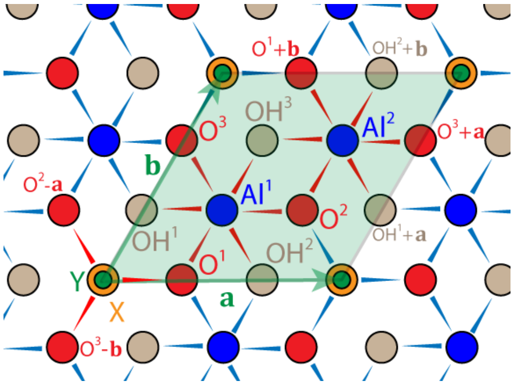

Supplementary Figure 1. The imogolite unit cell. Atoms and OH (CH_3) entities in the imogolite wall. The 10 atoms (entities) of the unit cell, highlighted in green, are labelled X, Y, Al^1, Al^2, O^1, O^2, O^3, OH^1, OH^2, OH^3. In this study X = Si or Ge and Y = CH_3. The position of other atoms (entities) can be figured out with translational symmetries along the unit cell vectors **a** and **b**. For instance, the position of the atom with label "$O^3 + \mathbf{a}$" is the position of atom "O^3" moved by **a**. Bounds in red are those considered in the total harmonic energy E_{geo}, while other bounds can also be figured out with translational symmetries along **a** and **b**. Angle bounds have not been displayed for the sake of clarity.

Supplementary Note 2. Reference values for bond lengths, angles and related harmonic constants

While reference lengths and angles are consistent within the literature, it appears that harmonic constants are not. Most spring constants we used were extracted from molecular dynamics studies based on CLAYFF force field. This force field gives valuable information on Si, Al and O based bonds, but it does not consider Ge nor CH_3 entities. The position of OH and CH_3 groups, considered as virtual atoms, is defined as the center of gravity of their electronic density. Force constants are taken equal to those for O and C atoms. Infra-red measurements were used in order to complete the harmonic constants data table. Some unknown harmonic constants have been roughly estimated through the relation $k = \mu(2\pi\sigma c)^2$ where μ is the reduced mass, k the harmonic constant and σ the wave number. For instance, knowing the stretching SiO and GeO wave numbers: $\sigma_{\nu\mathrm{SiO}}$ = 980 cm^{-1} [1], $\sigma_{\nu\mathrm{GeO}}$ = 810 cm^{-1} [2] from infrared experiments (IR) and from the relation $\frac{k^0_{\mathrm{GeO}}}{k^0_{\mathrm{SiO}}} = \frac{\mu_{\mathrm{GeO}}}{\mu_{\mathrm{SiO}}}\left(\frac{\sigma_{\nu\mathrm{GeO}}}{\sigma_{\nu\mathrm{SiO}}}\right)^2$, we figure out that the harmonic constants for GeO and SiO bonds are barely the same. Bond lengths, angles and harmonic constants used are reported in Supplementary Table 1, together with related sources.

Bonds	Length bound		Harmonic constant	
	Value (Å)	Source	Value ($J.m^{-2}$)	Source
**Si-O**<br>**Si-OH**	1.62<br>1.72	[3,4]	550	[3,5–7]
**Ge-O**<br>**Ge-OH**	1.73<br>1.83	[8]	550	Extrapolated from IR
**Si-C**<br>**Si-CH_3**	1.85<br>1.97	[9]	300	Extrapolated from IR
**Ge-C**<br>**Ge-CH_3**	1.95<br>2.07	[9]	300	Extrapolated from IR
**Al-O**<br>**Al-OH**	1.9<br>2.0	[10,11]	150	[3,7,12]
	**Angle**		**Angular harmonic constant**	
	**Value in degrees**	**Source**	**Value (10^{-20} $J.rad^{-2}$)**	**Source**
**O-Al-O**	90	Regular octahedron angle	70	[7,12]
**Al-O-Al**	90	Regular octahedron angle	14	[7]
**O-Si-O**	109.5	Regular tetrahedron angle	70	[6,7]
**O-Ge-O**	109.5	Regular tetrahedron angle	70	Taken equal to O-Si-O constant
**O-Si-C**	109.5	Regular tetrahedron angle	70	Taken equal to O-Si-O constant
**O-Ge-C**	109.5	Regular tetrahedron angle	70	Taken equal to O-Si-O constant
**Si-O-Al**	135	Link between regular octahedron and tetrahedron (geometrical calculation)	10	[7]
**Ge-O-Al**	135	Link between regular Octahedron and tetrahedron.	10	Taken equal to Si-O-Al constant

Supplementary Table 1. Bond lengths and angle values with the related harmonic constant value and the sources used.

Even if the values of spring constants are coarse estimations, multiple tests have been carried out and showed that the choice of these values barely affects result. Indeed, to quantify the impact of the choice of harmonic constants on the overall result, wide angle X-ray scattering (WAXS) was simulated with random harmonic force deviation α^i :

$$k^i_{\text{sample}} = k^i(1 + \alpha^i)\#(2)$$

where i refers to a bond or to a bond angle and k^i is the corresponding harmonic force constant given in Supplementary Table 1. α^i is a sample with random deviation from a normal distribution obtained using a standard deviation equal to 20%. 1000 random collections $\{\alpha_i\}$ were sampled. For each sample, the INT structure was relaxed by minimizing the geometrical energy and the WAXS diagram was calculated. Supplementary Figure 2 displays the envelope containing all calculated WAXS diagrams. Even for a large value of standard deviation (20%), the envelope is very narrow, so that a different choice of harmonic force constant will not affect data analysis.

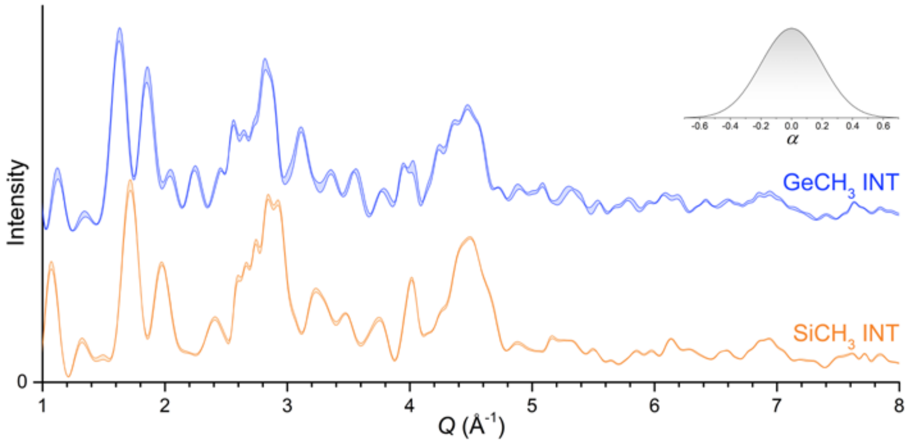

Supplementary Figure 2. Calculated WAXS diagrams with random harmonic force deviation. Calculated WAXS diagrams for $SiCH_3$ and $GeCH_3$ imogolite nanotubes (INT) with random harmonic force constant deviation α sampled from a normal distribution. The standard deviation of the distribution is equal to 20%.

Supplementary Note 3: WAXS diagrams' fitting process and results

The fitting procedure of WAXS experiments is the following:

0. Initialize values of the index *N*, of the inner radius R_i and of outer radius R_e.
1. Create atomic positions within the unit cell of a (*N*,*N*) nanotube with inner radius R_i, outer radius R_e and period *T*.
2. Minimize geometrical energy related to bond lengths and angles to obtain relaxed atomic coordinates.
3. Generate a nanotube of length 100 Å from the relaxed coordinates.
4. Calculate WAXS diagram with the Debye formula.
5. Compare the calculation and the experimental data through a chosen set of scattering features.
6. If the fit is suitable the algorithm stops; if not, it goes back to step 0 with another set of (R_i,R_e).

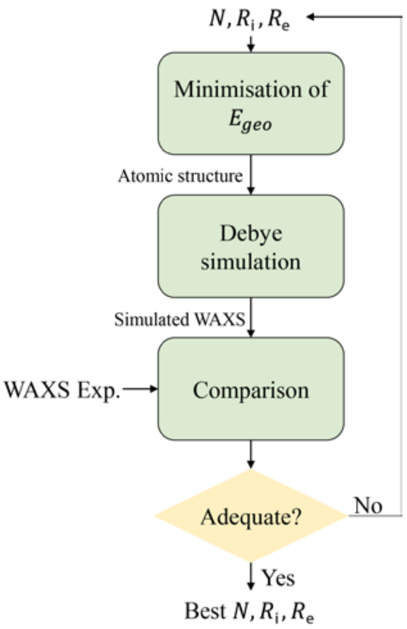

Supplementary Figure 1. Flowchart illustrating the WAXS fitting method.

Comparison between calculated and experimental data is performed on the positions of eight maxima and of one minimum of the experimental data, displayed in Supplementary Figures 4 and 5. They have been chosen to be easily identifiable by the comparison program because they appear well defined. As counter example, the shape of the scattered intensity around 2.6 – 2.8 $Å^{-1}$ is too complex to be a suitable reference point for a straightforward fitting procedure. Moreover, minima at wave-vectors smaller than 1 $Å^{-1}$ have not been taken into account because they are strongly dependent on porosity and water filling that are not the relevant parameters of the present structural analysis. Yet, this region is still useful for excluding some calculated data which appear suitable at high wave-vectors but which present strong discrepancies at small wave-vectors; see for example $GeCH_3$ INT for N = 13 in Supplementary Figure 4. Finally, consideration of the total harmonic energy E_{geo} (displayed on Supplementary Figures 4 and 5)

strengthens the comparison procedure since the lower E_{geo} is found to correspond to the structure giving the best agreement with WAXS data.

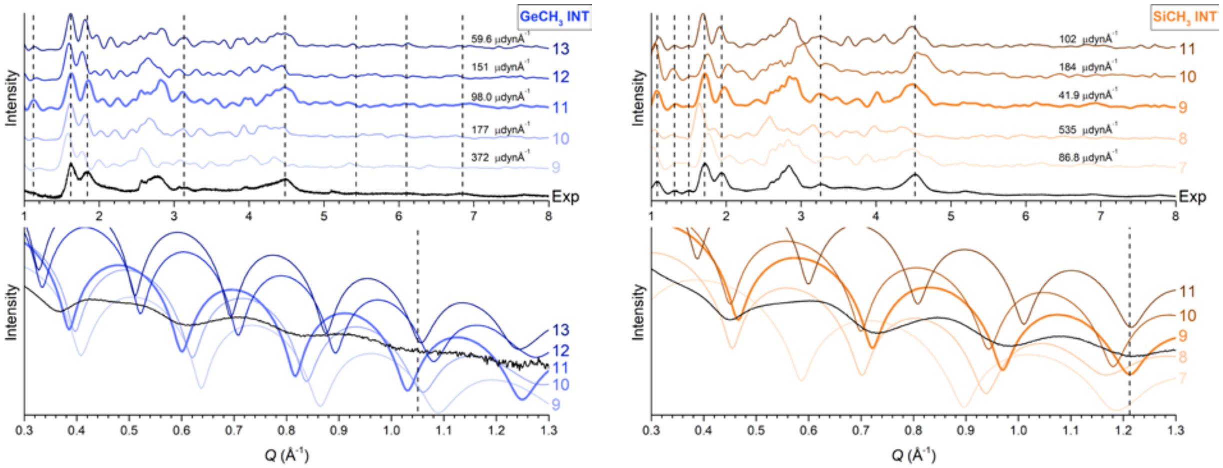

Supplementary Figure 4. Calculated WAXS diagrams of (*N*,*N*) m-INTs for different values of the *N* index. Calculated WAXS diagrams in best agreement with experimental ones, on left side, $GeCH_3$ imogolite nanotube powder and on right side, $SiCH_3$ one. Nanotube length was taken equal to 100 Å. The fit was performed using the positions of some maxima of the experimental WAXS diagram, displayed with dashed lines on top side and of the minimum displayed with a dashed line on down side. The value of the total harmonic energy E_{geo} is also indicated.

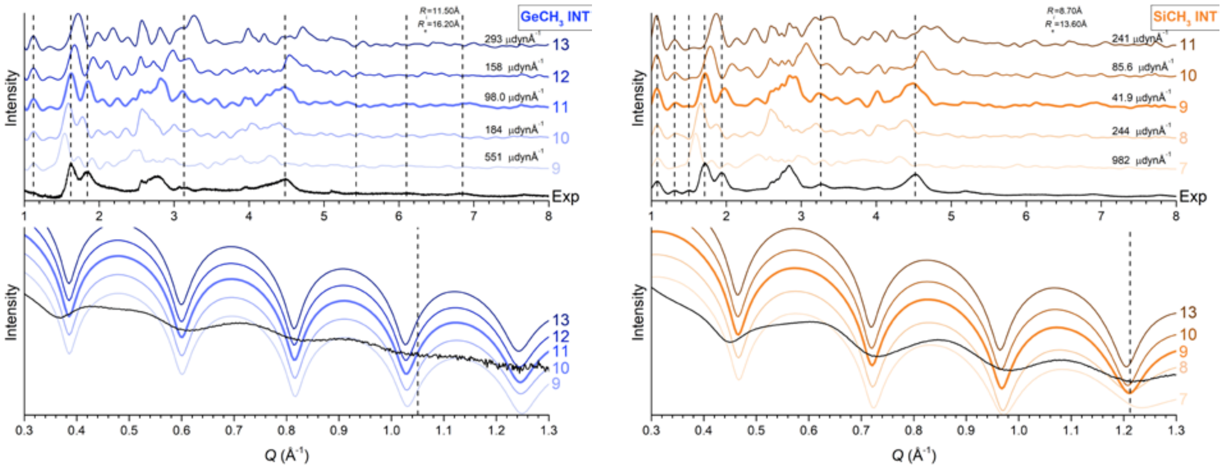

Supplementary Figure 5. Calculated WAXS diagrams of (*N*,*N*) m-INTs for different values of the *N* index and inner/outer radii fixed. Calculated WAXS diagrams for imogolite structure, $GeCH_3$ on left side and $SiCH_3$ on right side. R_i and R_e are fixed, for all *N* values, to radii obtained for the best fit of the experimental data in Supplementary Fig. 4 (R_i = 11.5 Å, R_i = 16.2 Å for N = 11 $GeCH_3$ INT and R_i = 8.7 Å, R_i = 13.6 Å for N = 9 for $SiCH_3$ INT). Nanotube length was taken equal to 100 Å. Dashed lines on top side and on down side point out maxima and, respectively, the minimum chosen to perform the fit. The value of the total harmonic energy E_{geo} is also indicated.

The structures corresponding to the best fits for $SiCH_3$ and $GeCH_3$ imogolite nanotubes are given in the Supplementary Table 2. Supplementary Figure 6 displays the cylindrical coordinates system in an imogolite nanotube.

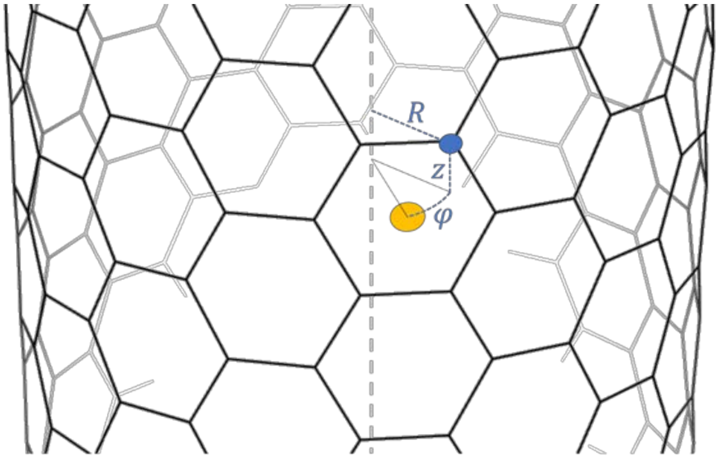

Supplementary Figure 6. Atom's cylindrical coordinates. Representation of cylindrical coordinates of an atom (in blue) of the imogolite nanotube. Si/Ge atom (in yellow) have $\varphi = 0°$ and $z = 0$.

**$SiCH_3$ INT**	Atom	OH			Al		O			Si	CH_3	N	$\gamma(°)$
	$R(Å)$	13.6*			12.4		11.4			10.8	8.8*	9*	66.1
	$\varphi(°)$	7.4	12.6	20	13.4	26.6	6.5	20	13.5	0*	0*	$T(Å)$	$a(Å)$
	$z(Å)$	-0.8	1.6	-0.7	0	0	0.8	0.9	-1.6	0*	0*	4.89*	4.48
**$GeCH_3$ INT**	Atom	OH			Al		O			Ge	CH_3	N	$\gamma(°)$
	$R(Å)$	16.2*			15.1		14.0			13.6	11.6*	11*	65.0
	$\varphi(°)$	5.7	10.6	16.4	10.9	21.8	5.7	16.4	10.7	0*	0*	$T(Å)$	$a(Å)$
	$z(Å)$	-0.8	1.7	-0.7	0	0	0.9	0.8	-1.6	0*	0*	4.95*	4.60

Supplementary Table 2. Atoms coordinates after the energy minimization of $SiCH_3$ and $GeCH_3$ INT. Cylindrical coordinates after the energy minimization in $SiCH_3$ and $GeCH_3$ INT unit cells and values of the index N and of the period T. The stars indicate fixed values: the period T deduced from the position of asymmetrical 00l Bragg peaks, R_i and R_e as parameters of the fitting procedure, (φ,z) of Si/Ge as origin of the cylindrical coordinates and (φ,z) of CH_3 assuming that the scattering entity is radially lined up with Si/Ge. The refined values of the unit cell parameters, namely the modulus a of the unit cell vectors in Supplementary fig. 1 and the angle γ between them, are given in the last column.

Supplementary Note 4: Imogolite wall thickness

The imogolite wall thickness Δz can be estimated by geometrical consideration on an alumino-silicate/germanate planar sheet. For regular tetrahedra and octahedra as displayed in Supplementary Figure 7, we can easily show:

$$\Delta z = \Delta z_{\mathrm{CH_3-X}} + \Delta z_{\mathrm{X-O}} + \Delta z_{\mathrm{O-Al}} + \Delta z_{\mathrm{Al-OH}} = d_{\mathrm{CH_3-X}} + \frac{d_{\mathrm{X-O}}}{3} + \frac{d_{\mathrm{Al-O}}}{\sqrt{3}} + \frac{d_{\mathrm{Al-OH}}}{\sqrt{3}} \#(3)$$

where $\Delta z_{\mathrm{A-B}}$ is the distance between layers defined by entities A and by entities B and $d_{\mathrm{A-B}}$ is the bond length between A and B. Taking the values given in Supplementary Table 1, we estimate the imogolite wall thickness at 4.7 Å for $SiCH_3$ and 4.84 Å for $GeCH_3$. These values are in good agreement with the thicknesses found through the fitting procedure: 4.8 Å for $SiCH_3$ INT and 4.6 Å for $GeCH_3$ INT. These differences come from the distortion induced by the wrapping of the atomic structure.

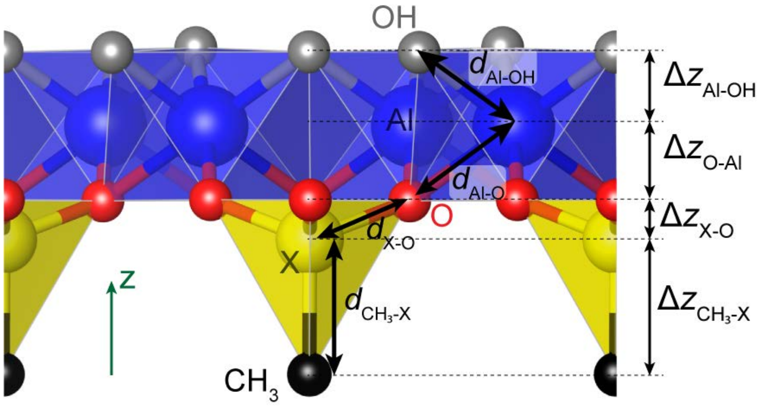

Supplementary Figure 7. The Imogolite wall thickness. Sectional view of a methyl imogolite planar sheet (X = Si for $SiCH_3$ INT and X = Ge for $GeCH_3$ INT). The bond length between scattering entities A and B is given by $d_{\mathrm{A-B}}$. The distance between two successive layers A and B is given by $\Delta z_{\mathrm{A-B}}$.

Supplementary Note 5: Effect of the nanotubes' length on the calculated WAXS diagrams

Decrease of the nanotube length results in a smoothing of the whole WAXS diagram as is shown in Supplementary Figure 8. We chose here to compute nanotubes with a given length and not to introduce a distribution in lengths. Indeed, it would have been too ambitious to introduce additional parameters featuring the length distribution without over-parameterizing the fitting of WAXS experiment.

Lengths $L = 20 \times T$, where T is the period, give the best agreement between calculated and measured oscillations after the first asymmetrical peak around 2.5 – 2.6 $Å^{-1}$ for $SiCH_3$ and $GeCH_3$ INTs' powders. We thus considered a correlation length of about 100 Å for both types of nanotubes (T = 4.95 Å for $GeCH_3$ INT and 4.89 Å for $SiCH_3$ INT).

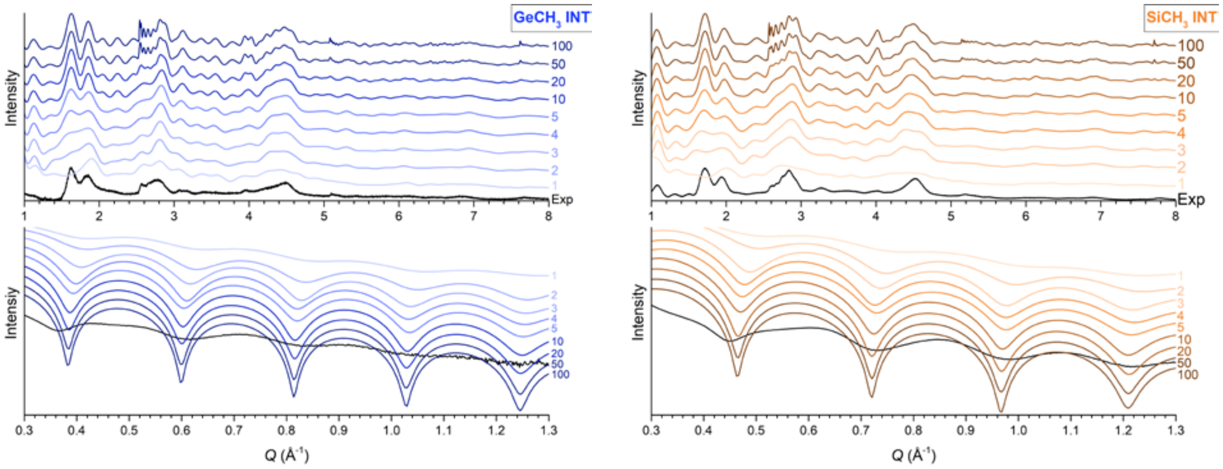

Supplementary Figure 8. Calculated WAXS diagrams for different nanotube's lengths. Calculated WAXS diagrams of powders of imogolite nanotubes with different lengths $L = n \times T$, where T is the period of the, n value being given on the right of each curve. The atomic coordinates used are the refined ones of Supplementary Table 2. Left: $GeCH_3$ powder, right: $SiCH_3$ powder.

Supplementary Note 6: Distribution of chiral indices

A possible distribution of chiral indices (N,M) corresponding to radii and chiral angles (defined as the angle χ between the basis vector **a** and the chiral vector $\mathbf{C}_{NM} = N\mathbf{a} + M\mathbf{b}$ in Figure 2 in the manuscript) around the ones we find thanks to our fitting procedure cannot be fitted on the basis of experimental diagrams. By adding further parameters into the fitting procedure, we would over-parameterize the model. One can only conclude, as discussed below, that a majority of $SiCH_3$ (resp. $GeCH_3$) imogolite nanotubes have (9,9) indices (resp. (11,11) indices).

Supplementary Figure 9 displays the scattered intensity from a powder with a proportion p of nanotubes with chiral indices (N, N) and a proportion of (1-p)/2 of nanotubes with chiral indices equal to (N-1, N-1) or (N+1, N+1):

$$I(Q) = pI_N(Q) + \frac{(1-p)}{2}\left(I_{N-1}(Q) + I_{N+1}(Q)\right) \# (4)$$

where $I_{N-1}(Q)$, $I_N(Q)$ and $I_{N+1}(Q)$ are shown in Supplementary Figure 4. One cannot exclude the occurrence of a small proportion of (N-1, N-1) and (N+1, N+1) nanotubes together with (N, N) nanotubes (calculated intensity for p = 0.8 is in rather good agreement with experimental diagrams). Based on high-resolution cryo-electron microscopy, previous studies[2,13] estimated that the radius of imogolite could fluctuate by ±1-2Å, which would correspond to the presence of a small proportion of (N-1, N-1) and (N+1, N+1) nanotubes. As stated from Supplementary Figure 9 for radius distribution, a distribution in chiral angles χ around the one corresponding to the armchair one (χ = 30°) cannot be ruled out. However, rapid oscillations on the high-Q tails of 002 peaks measured experimentally being rather sensitive to chiral angle variations[14], such distribution should be narrow, like the diameter distribution if any.

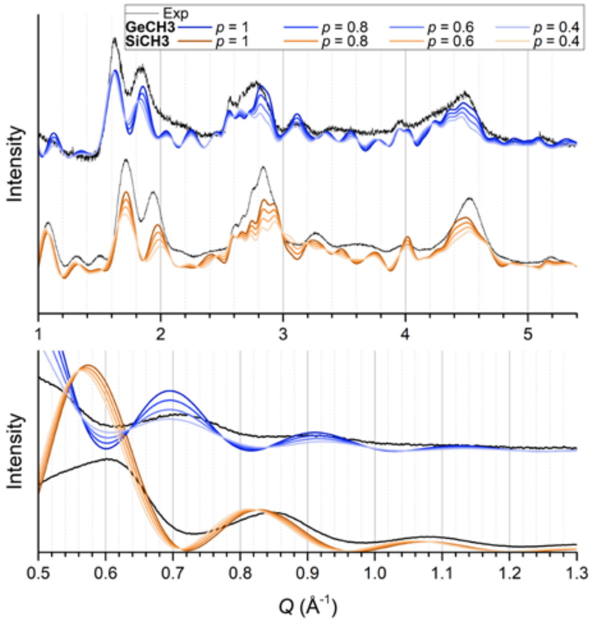

Supplementary Figure 9. Calculated WAXS diagrams for a powder of m-INTs with a distribution in radius. Weighted sum of the calculated intensities for (*N*-1, *N*-1), (*N*, *N*) and (*N*+1, *N*+1) methylated imogolite nanotubes.

Supplementary Note 7: Calculated WAXS diagrams of DFT-relaxed structures

Supplementary Figure 10 displays a good agreement between experimental and calculated WAXS diagrams for the DFT-relaxed armchair structures with N = 11 for $GeCH_3$ and N = 9 for $SiCH_3$. In comparison to the curve extracted from the wide angle fitting procedure and the simple minimization of the geometrical energy, the agreement is highly satisfactory at wide angle but the minima at smaller wave-vector are somehow misaligned. This misalignment indicates that the radii of relaxed tube are not in total agreement with the experimental data. This can be explained by the fact that ab-initio calculations have been carried out in vacuum so that the role of the solvent was not considered. This last point goes beyond the scope of this article.

Supplementary Figure 11 displays stark divergence between experimental WAXS diagrams at wide angle and those computed for the DFT-relaxed zig-zag structures. It confirms that the inner-wall atomic organization for a zigzag chirality is incompatible with experimental WAXS diagrams.

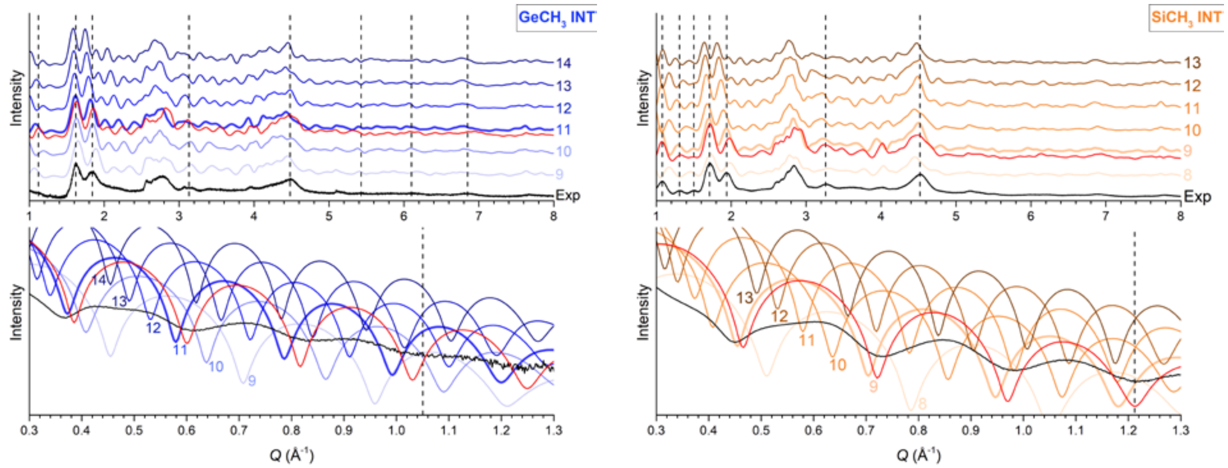

Supplementary Figure 10. Calculated WAXS diagrams for DFT-optimized armchair imogolite structures. Calculated WAXS diagrams for the armchair (*N*,*N*) imogolite structures DFT-PBE optimized at a fixed value of the period *T* (4.95 Å for $GeCH_3$ INT and 4.88 Å for $SiCH_3$ INT). The results are displayed for a range of *N* values around the one that fits the experimental curve. *N* values are indicated on the right of the calculated curves. Calculated diagrams for the PBE-D3 optimized structures are not shown as indistinguishable from the PBE results. Experimental diagrams are shown in dark. The red curve is the best result from the wide angle fitting procedure based on the minimization of the geometrical energy and on the WAXS fitting procedure described above.

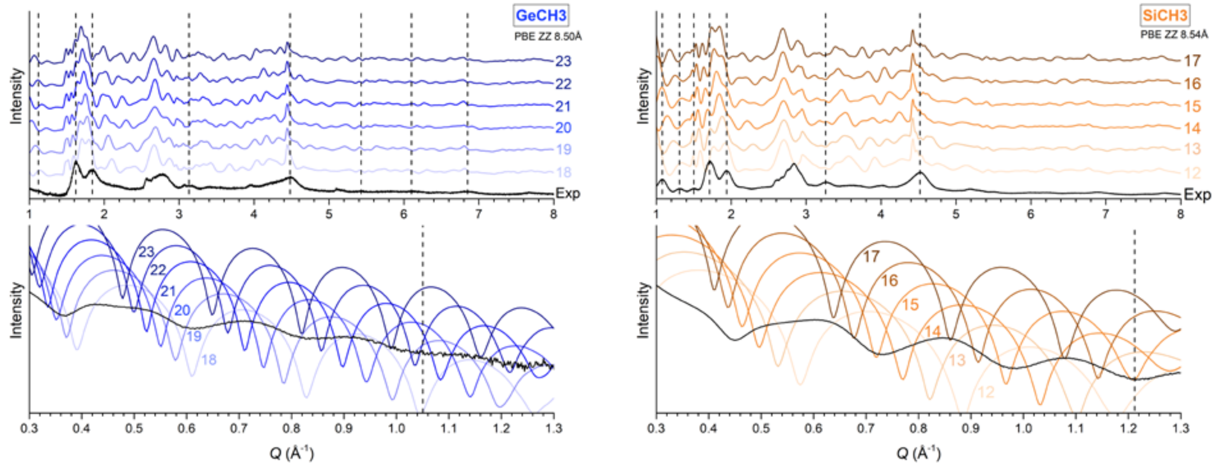

Supplementary Figure 11. Calculated WAXS diagrams for DFT-optimized zigzag imogolite structures. Calculated WAXS diagrams for the DFT-PBE optimized zigzag (*N*,0) imogolite structures. *N* values are indicated on the right of the calculated curves. The periodicity has also been optimized at PBE level (8.50 Å for $GeCH_3$ INT, 8.54 Å for $SiCH_3$ INT). Experimental diagrams are shown in dark.

Supplementary Note 8: Analysis of bond lengths and angles in DFT-optimized structures

Bond lengths and angles corresponding to the PBE and PBE-D3 DFT optimized structures for armchair (AC) (*N*,*N*) and zigzag (ZZ) (*N*,0) nanotubes are displayed in Supplementary Figures 12 to 21. Atoms' labelling refers to Figure 1 of the manuscript. For both $SiCH_3$ and $GeCH_3$ INTs, the differences between the average H_1-C_2, C_2-Si_3(Ge_3) and Si_3(Ge_3)-O_4 bond distances for the computed AC and ZZ $E/2N$ minima are found to be smaller than 5×10^{-3} Å (Supplementary Figures 12 to 14). Conversely, for $SiCH_3$ nanotubes, up to one order of magnitude larger differences (in the order of 10^{-2} Å) are found for the O_4-Al_5, Al_5-O_6 and O_6-H_7 bonds of the AC and ZZ structures (Supplementary Figures 15 to 17). Specifically, the armchair rolling is computed to lead to O_4-Al_5 and Al_5-O_6 bond lengths closer to the value computed for planar (hydroxylated) imogolite sheets at PBE level (1.92 Å [15]), indicative of reduced strain of the gibbsite layer for AC nanotubes with respect to ZZ ones. In contrast to the results for $SiCH_3$ nanotubes, we find very limited differences (<5×10^{-3} Å) also in the O_4-Al_5, Al_5-O_6 and O_6-H_7 bonds between the AC and ZZ $E/2N$ minima for the $GeCH_3$ systems (Supplementary Figures 15 to 17), which prompts for study of the distortions in the bond-angles of the gibbsite layer.

The inner O_4-Al_5-O_4 angles are found to be substantially closer (up to over 2 degrees) to the ideal octahedron values (90 degrees) for the $SiCH_3$ AC nanotubes (Supplementary Figure 19). Conversely, progressively smaller (<1 degree) differences between AC and ZZ structures are computed for the outer O_6-Al_5-O_6 and intra-wall O_4-Al_5-O_6 angles (Supplementary Figures 20 and 21), suggesting that these structural parameters do not majorly contribute to the energy favorability of AC rolling. It is worth noting that, contrary to the $SiCH_3$ case, AC or ZZ rolling of the $GeCH_3$ nanotubes lead to negligible differences in the average O_4-Al_5-O_4 angle (Supplementary Figure 19). However, the average O_6-Al_5-O_6 angle for the GeCH3 AC $E/2N$ minimum is roughly 1 degree closer to the ideal 90 degrees value than for the ZZ $E/2N$ minimum (Supplementary Figure 20), contributing to the energetic stabilization of the AC structure.

Thus, it appears that the stabilization of methylated imogolite nanotubes mainly occurs in the gibbsite layer spanning the O_4-Al_5-O_6 atoms, with different mechanisms for $SiCH_3$ and $GeCH_3$ systems. Whereas for $GeCH_3$ INTs, the AC configuration becomes energetically favored through the reduction of the strain in the (O_6-Al_5-O_6) angles in the octahedral layer, for $SiCH_3$ the stabilization arises from reduced distortions of the O_4-Al_5 and Al_5-O_6 bonds, and of the O_4-Al_5-O_4 angle.

The computed bond distances for the inner pendant groups [H_1-C_2, C_2-Si_3(Ge_3), and Si_3(Ge_3)-O_4 bonds] are minimally affected by AC or ZZ rolling, with deviations smaller than 5×10^{-3} Å (Supplementary Figures 12 to 14). The latter results clearly indicate that relaxation of the inner methyl groups and adjacent C_2-Si_3(Ge_3)-O_4 tetrahedron does not play any major role in lowering the energy of AC nanotubes with respect to ZZ analogs.

The over 0.1 Å shorter hydrogen bonding distances on the outer surface of the AC $SiCH_3$ nanotubes with respect to the ZZ ones (Supplementary Figure 18) provides further energetic favorability to AC rolling over the ZZ geometry. The substantially smaller (<0.02 Å) differences in hydrogen bonding distances on the outer surface of AC and ZZ $GeCH_3$ nanotubes indicate this additional mechanism of stabilization is not present for larger-diameter (reduced curvature) nanotubes, reiterating that depending on the presence of either Si or Ge the geometric origins of AC stabilization are different.

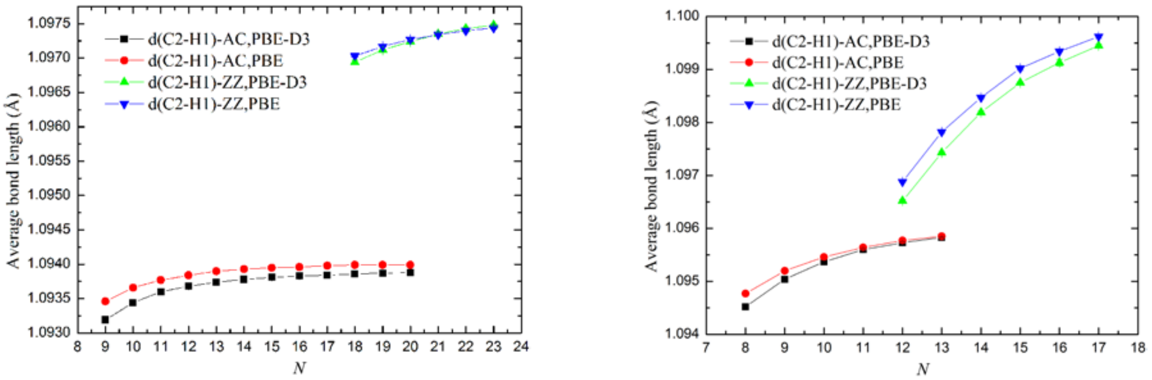

Supplementary Figure 12. H_1-C_2 bond lengths. Computed average H_1-C_2 ($d_{H1\text{-}C2}$) bond lengths (Å), with standard deviation within the symbols size, for the $GeCH_3$ (left) and $SiCH_3$ (right) nanotubes at PBE and PBE-D3 level. Error bars report the standard deviation of the displayed average values. Averages have been computed over all the relevant bond-distances of the given nanotube model.

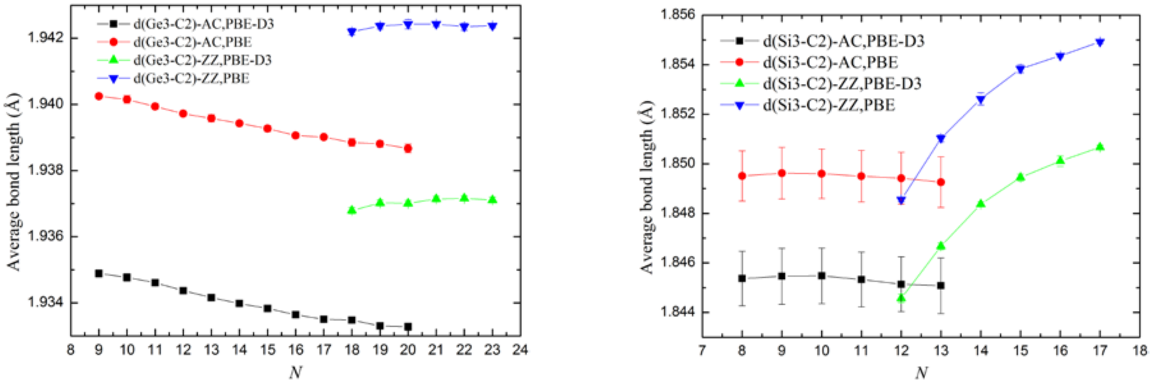

Supplementary Figure 13. C_2-Si_3(Ge_3) bond lengths. Computed average C_2-Si_3(Ge_3) ($d_{C2\text{-}Si3}$ and $d_{C2\text{-}Ge3}$) bond lengths (Å) with standard deviation for the $GeCH_3$ (left) and $SiCH_3$ (right) nanotubes at PBE and PBE-D3 level. Error bars report the standard deviation of the displayed average values. Averages have been computed over all the relevant bond-distances of the given nanotube model.

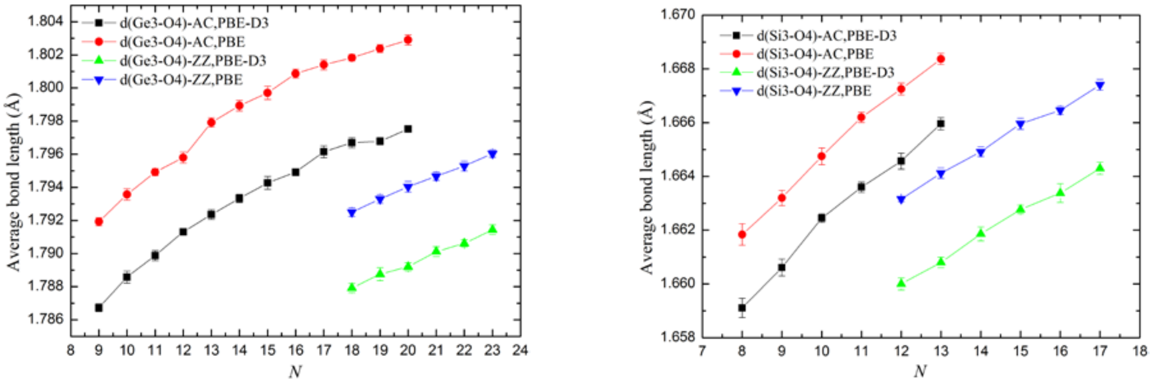

Supplementary Figure 14. Si_3(Ge_3)-O_4 bond lengths. Computed average Si_3(Ge_3)-O_4 ($d_{Si3\text{-}O4}$ and $d_{Ge3\text{-}O4}$) bond lengths (Å) with standard deviation for the $GeCH_3$ (left) and $SiCH_3$ (right) nanotubes at PBE and PBE-D3 level. Error bars report the standard deviation of the displayed average values. Averages have been computed over all the relevant bond-distances of the given nanotube model.

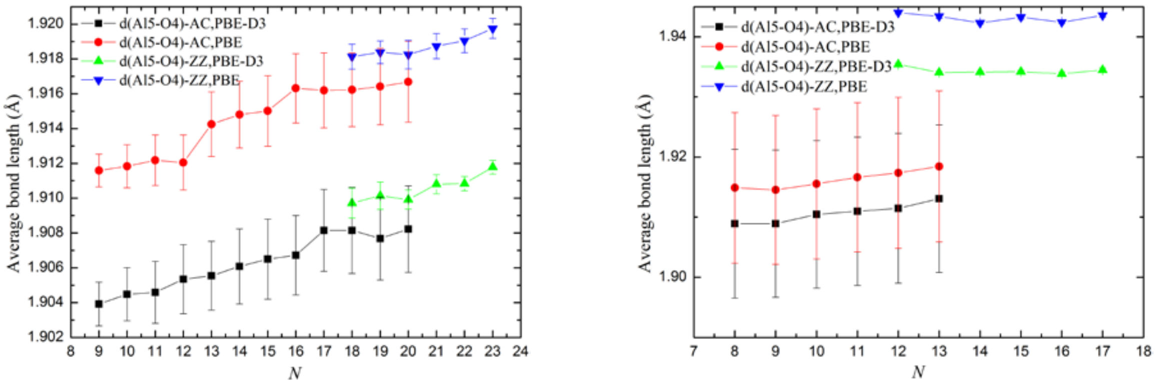

Supplementary Figure 15. O_4-Al_5 bond lengths. Computed average O_4-Al_5 (d_{O4-Al5}) bond lengths (Å) with standard deviation for the $GeCH_3$ (left) and $SiCH_3$ (right) nanotubes at PBE and PBE-D3 level. Error bars report the standard deviation of the displayed average values. Averages have been computed over all the relevant bond-distances of the given nanotube model.

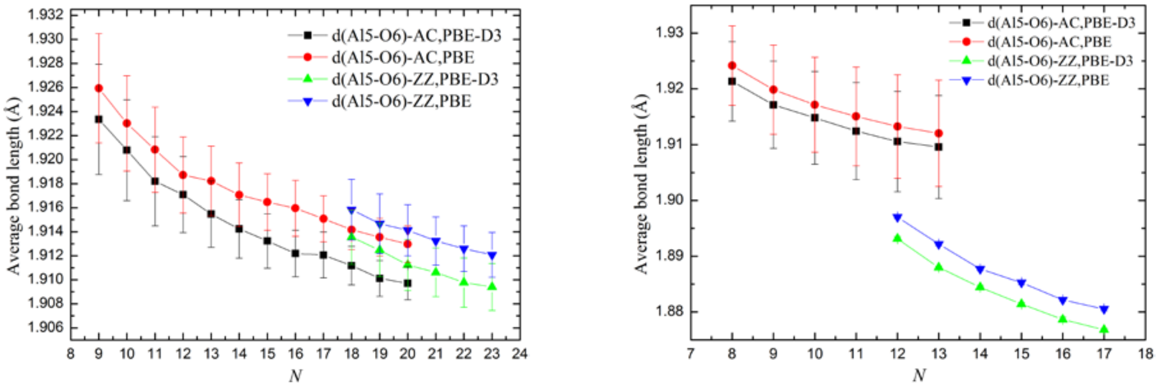

Supplementary Figure 16. Al_5-O_6 bond lengths. Computed average Al_5-O_6 (d_{Al5-O6}) bond lengths (Å) with standard deviation for the $GeCH_3$ (left) and $SiCH_3$ (right) nanotubes at PBE and PBE-D3 level. Error bars report the standard deviation of the displayed average values. Averages have been computed over all the relevant bond-distances of the given nanotube model.

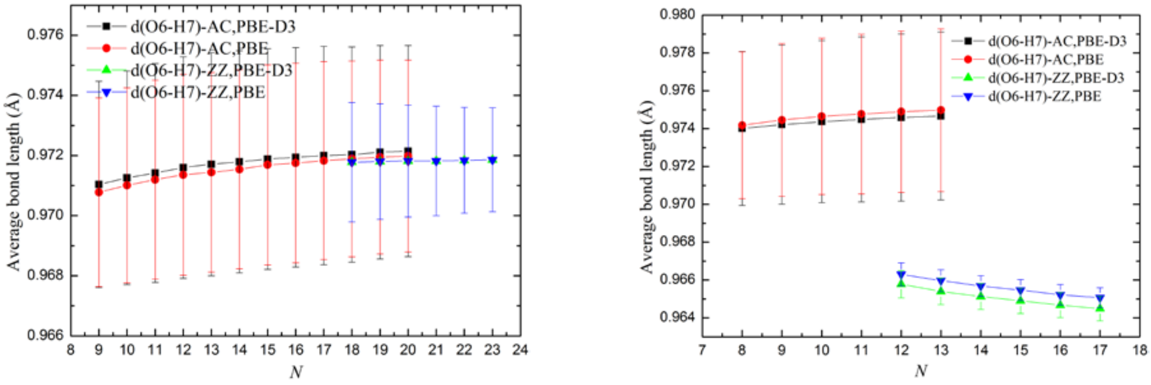

Supplementary Figure 17. O_6-H_7 bond lengths. Computed average O_6-H_7 (d_{O6-H7}) bond lengths (Å) with standard deviation for the $GeCH_3$ (left) and $SiCH_3$ (right) nanotubes at PBE and PBE-D3 level. Error bars report the standard deviation of the displayed average values. Averages have been computed over all the relevant bond-distances of the given nanotube model.

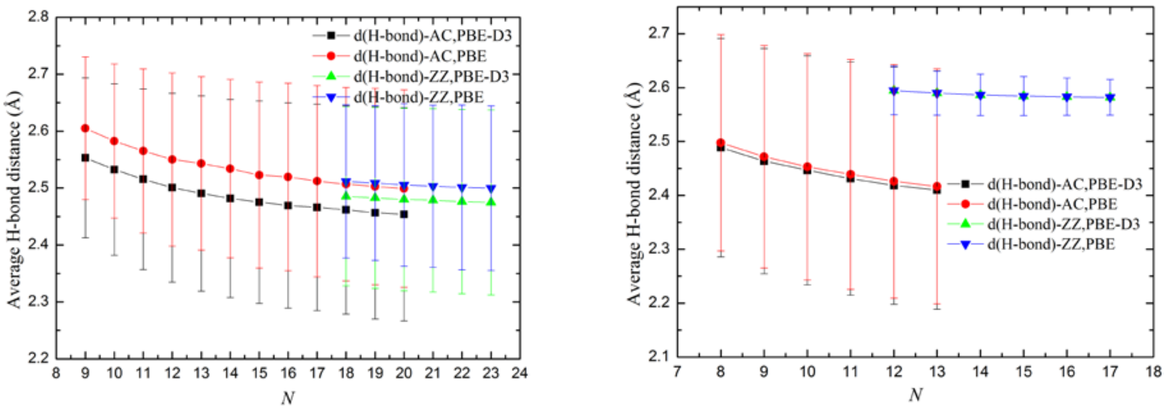

Supplementary Figure 18. Outer surface H-bond distances. Computed average outer surface H-bond distances [*d*(H-bond), Å] with standard deviation for the $GeCH_3$ (left) and $SiCH_3$ (right) nanotubes at PBE and PBE-D3 level. Error bars report the standard deviation of the displayed average values. Averages have been computed over all the relevant bond-distances of the given nanotube model.

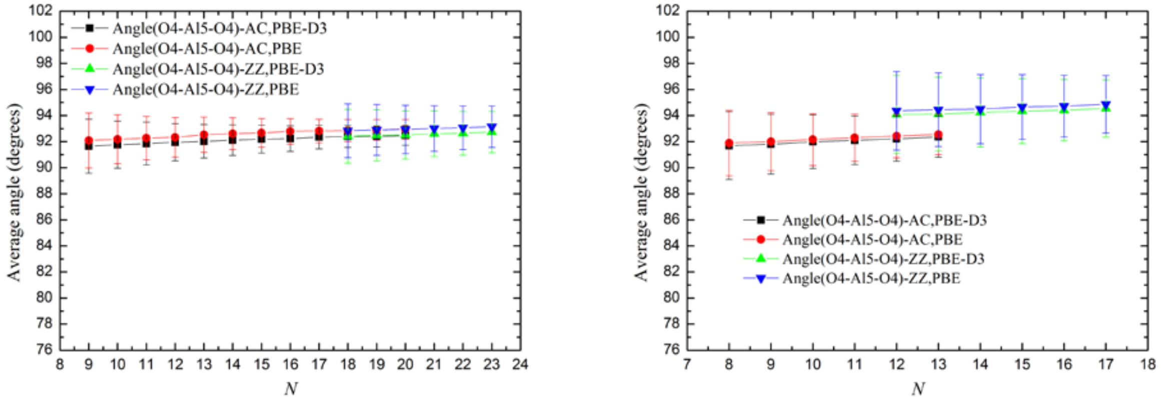

Supplementary Figure 19. O_4-Al_5-O_4 angles. Computed average O_4-Al_5-O_4 angles (degrees) with standard deviation for the $GeCH_3$ (left) and $SiCH_3$ (right) nanotubes at PBE and PBE-D3 level. Error bars report the standard deviation of the displayed average values. Averages have been computed over all the relevant bond-angles of the given nanotube model.

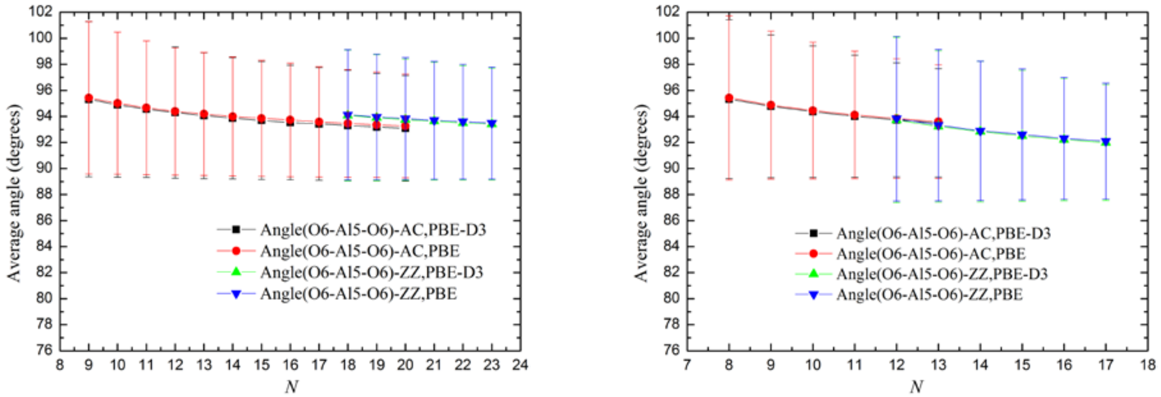

Supplementary Figure 20. O_6-Al_5-O_6 angles. Computed average O_6-Al_5-O_6 angles (degrees) with standard deviation for the $GeCH_3$ (left) and $SiCH_3$ (right) nanotubes at PBE and PBE-D3 level. The increased standard deviations with respect to Supplementary Figure 19 stem from the larger deviations in the value of the O_6-Al_5-O_6 angles depending on whether the given Al_5-O_6 bond runs along the nanotube axis or circumference, see also Supplementary Figure 1 and Figure 1 in the manuscript. Error bars report the standard deviation of the displayed average values. Averages have been computed over all the relevant bond-angles of the given nanotube model.

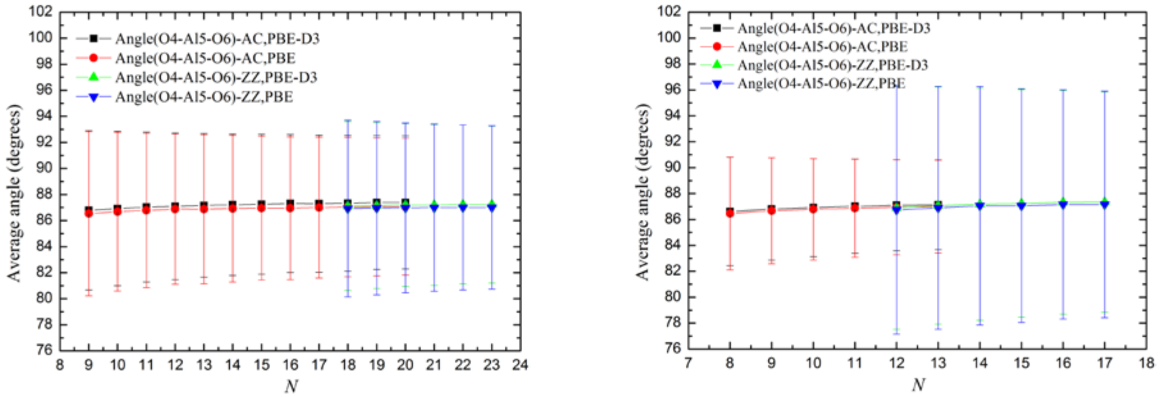

Supplementary Figure 21. O_4-Al_5-O_6 angles. Computed average O_4-Al_5-O_6 angles (degrees) with standard deviation for the $GeCH_3$ (left) and $SiCH_3$ (right) nanotubes at PBE and PBE-D3 level. The increased standard deviations with respect to Supplementary Figure 19 stem from the larger deviations in the value of the O_4-Al_5-O_6 angles depending on whether the given Al_5-O_6 bond run along the nanotube axis or circumference, see also Supplementary Figure 1 and Figure 1 in the manuscript. Error bars report the standard deviation of the displayed average values. Averages have been computed over all the relevant bond-angles of the given nanotube model.

Supplementary Note 9: Atomic radii of DFT-optimized structures

System	$R(H_1)$	$R(C_2)$	$R(Si_3)$	$R(O_4)$	R(Al5)	$R(O_6)$	$R(H_7)$
(12,0)	6.307±0.160	6.648±0.003	8.485±0.003	9.129±0.020	10.092±0.003	11.176±0.027	11.767±0.005
(13,0)	7.060±0.122	7.409±0.007	9.252±0.007	9.893±0.019	10.859±0.007	11.945±0.025	12.534±0.007
(14,0)	7.813±0.099	8.166±0.007	10.013±0.007	10.651±0.018	11.620±0.008	12.708±0.024	13.294±0.008
(15,0)	8.604±0.086	8.961±0.017	10.810±0.016	11.444±0.023	12.414±0.017	13.502±0.027	14.087±0.017
(16,0)	9.366±0.074	9.725±0.002	11.576±0.002	12.207±0.014	13.179±0.002	14.268±0.020	14.851±0.003
(17,0)	10.167±0.075	10.528±0.032	12.380±0.032	13.008±0.035	13.980±0.033	15.069±0.038	15.651±0.033

Supplementary Table 3. Radii in ZZ $SiCH_3$ PBE-optimized nanotubes. Computed average atomic radii with standard deviation for the ZZ $SiCH_3$ nanotubes at PBE level.

System	$R(H_1)$	$R(C_2)$	$R(Si_3)$	$R(O_4)$	R(Al5)	$R(O_6)$	$R(H_7)$
(12,0)	6.270±0.173	6.607±0.005	8.438±0.005	9.082±0.022	10.047±0.005	11.131±0.027	11.714±0.006
(13,0)	7.009±0.134	7.353±0.011	9.191±0.011	9.832±0.022	10.800±0.011	11.887±0.027	12.467±0.011
(14,0)	7.775±0.108	8.124±0.006	9.966±0.006	10.604±0.019	11.574±0.006	12.662±0.023	13.240±0.009
(15,0)	8.546±0.093	8.899±0.013	10.743±0.013	11.377±0.021	12.349±0.013	13.437±0.025	14.014±0.018
(16,0)	9.311±0.082	9.667±0.020	11.513±0.020	12.144±0.026	13.117±0.021	14.206±0.028	14.782±0.025
(17,0)	10.099±0.078	10.456±0.030	12.304±0.030	12.931±0.034	13.906±0.030	14.995±0.035	15.570±0.032

Supplementary Table 4. Radii in ZZ $SiCH_3$ PBE-D3-optimized nanotubes. Computed average atomic radii with standard deviation for the ZZ $SiCH_3$ nanotubes at PBE-D3 level.

System	$R(H_1)$	$R(C_2)$	$R(Si_3)$	$R(O_4)$	R(Al5)	$R(O_6)$	$R(H_7)$
(8,8)	7.629±0.104	7.975±0.009	9.818±0.009	10.468±0.042	11.476±0.020	12.536±0.032	13.045±0.383
(9,9)	8.955±0.092	9.305±0.013	11.150±0.012	11.793±0.038	12.806±0.021	13.868±0.034	14.374±0.384
(10,10)	10.309±0.084	10.663±0.028	12.509±0.027	13.146±0.043	14.161±0.032	15.225±0.042	15.729±0.385
(11,11)	11.672±0.075	12.028±0.020	13.874±0.020	14.506±0.036	15.523±0.025	16.588±0.037	17.091±0.385
(12,12)	13.030±0.068	13.388±0.008	15.235±0.008	15.862±0.029	16.880±0.018	17.947±0.032	18.448±0.384
(13,13)	14.399±0.067	14.760±0.026	16.607±0.026	17.230±0.037	18.249±0.031	19.317±0.041	19.817±0.385

Supplementary Table 5. Radii in AC $SiCH_3$ PBE-optimized nanotubes. Computed average atomic radii with standard deviation for the AC $SiCH_3$ nanotubes at PBE level.

System	$R(H_1)$	$R(C_2)$	$R(Si_3)$	$R(O_4)$	R(Al5)	$R(O_6)$	$R(H_7)$
(8,8)	7.557±0.110	7.898±0.014	9.736±0.013	10.386±0.043	11.396±0.022	12.456±0.035	12.962±0.384
(9,9)	8.878±0.098	9.224±0.023	11.064±0.022	11.707±0.043	12.720±0.028	13.784±0.039	14.287±0.385
(10,10)	10.234±0.084	10.583±0.011	12.425±0.010	13.061±0.034	14.077±0.019	15.142±0.034	15.643±0.384
(11,11)	11.574±0.077	11.926±0.015	13.768±0.014	14.399±0.033	15.417±0.021	16.484±0.036	16.984±0.384
(12,12)	12.917±0.074	13.271±0.022	15.114±0.022	15.741±0.036	16.760±0.028	17.828±0.039	18.326±0.385
(13,13)	14.293±0.067	14.649±0.017	16.492±0.017	17.114±0.031	18.134±0.023	19.203±0.037	19.701±0.384

Supplementary Table 6. Radii in AC $SiCH_3$ PBE-D3-optimized nanotubes. Computed average atomic radii with standard deviation for the AC $SiCH_3$ nanotubes at PBE-D3 level.

System	$R(H_1)$	$R(C_2)$	$R(Si_3)$	$R(O_4)$	R(Al5)	$R(O_6)$	$R(H_7)$
(18,0)	10.913±0.063	11.250±0.010	13.189±0.010	13.894±0.024	14.899±0.010	15.966±0.033	16.483±0.267
(19,0)	11.704±0.059	12.042±0.013	13.982±0.013	14.684±0.025	15.691±0.014	16.758±0.034	17.271±0.268
(20,0)	12.488±0.059	12.828±0.022	14.768±0.022	15.466±0.031	16.474±0.023	17.542±0.037	18.052±0.270
(21,0)	13.288±0.053	13.628±0.014	15.569±0.014	16.264±0.024	17.273±0.015	18.341±0.032	18.848±0.270
(22,0)	14.084±0.052	14.425±0.019	16.366±0.019	17.059±0.027	18.068±0.020	19.136±0.034	19.641±0.272
(23,0)	14.892±0.048	15.235±0.014	17.176±0.014	17.866±0.023	18.876±0.015	19.944±0.031	20.447±0.272

Supplementary Table 7. Radii in ZZ $GeCH_3$ PBE-optimized nanotubes. Computed average atomic radii with standard deviation for the ZZ $GeCH_3$ nanotubes at PBE level.

System	$R(H_1)$	$R(C_2)$	$R(Si_3)$	$R(O_4)$	R(Al5)	$R(O_6)$	$R(H_7)$
(18,0)	10.824±0.070	11.157±0.011	13.091±0.011	13.797±0.026	14.807±0.011	15.874±0.033	16.374±0.274
(19,0)	11.614±0.072	11.948±0.032	13.883±0.032	14.585±0.039	15.597±0.033	16.664±0.045	17.161±0.277
(20,0)	12.387±0.062	12.723±0.018	14.658±0.017	15.358±0.027	16.371±0.018	17.438±0.035	17.933±0.277
(21,0)	13.192±0.059	13.529±0.017	15.464±0.017	16.161±0.027	17.174±0.018	18.242±0.033	18.735±0.277
(22,0)	13.975±0.053	14.313±0.015	16.248±0.015	16.943±0.024	17.957±0.015	19.025±0.032	19.516±0.278
(23,0)	14.785±0.060	15.124±0.033	17.059±0.033	17.751±0.038	18.765±0.034	19.834±0.043	20.323±0.280

Supplementary Table 8. Radii in ZZ $GeCH_3$ PBE-D3-optimized nanotubes. Computed average atomic radii with standard deviation for the ZZ $GeCH_3$ nanotubes at PBE-D3 level.

System	$R(H_1)$	$R(C_2)$	$R(Si_3)$	$R(O_4)$	R(Al5)	$R(O_6)$	$R(H_7)$
(9,9)	9.012±0.081	9.338±0.012	11.274±0.011	11.981±0.024	12.992±0.013	14.046±0.026	14.544±0.306
(10,10)	10.372±0.075	10.702±0.020	12.639±0.020	13.339±0.027	14.353±0.021	15.408±0.030	15.902±0.307
(11,11)	11.737±0.071	12.069±0.027	14.006±0.027	14.700±0.030	15.717±0.028	16.774±0.035	17.264±0.308
(12,12)	13.091±0.062	13.425±0.019	15.363±0.018	16.053±0.022	17.071±0.020	18.130±0.028	18.616±0.308
(13,13)	14.519±0.074	14.855±0.051	16.793±0.051	17.477±0.052	18.495±0.051	19.554±0.055	20.040±0.311
(14,14)	15.901±0.054	16.238±0.018	18.176±0.018	18.855±0.021	19.875±0.020	20.935±0.027	21.418±0.307
(15,15)	17.274±0.050	17.612±0.015	19.550±0.014	20.226±0.017	21.248±0.017	22.307±0.025	22.788±0.307
(16,16)	18.688±0.043	19.027±0.014	20.965±0.014	21.638±0.016	22.659±0.016	23.719±0.024	24.199±0.307
(17,17)	20.051±0.045	20.391±0.014	22.329±0.014	22.999±0.016	24.022±0.016	25.083±0.024	25.560±0.306
(18,18)	21.422±0.046	21.763±0.021	23.701±0.021	24.369±0.022	25.392±0.023	26.453±0.028	26.930±0.306
(19,19)	22.801±0.045	23.142±0.023	25.080±0.023	25.746±0.024	26.770±0.024	27.832±0.029	28.307±0.307
(20,20)	24.184±0.035	24.527±0.013	26.465±0.012	27.129±0.014	28.153±0.014	29.215±0.021	29.689±0.306

Supplementary Table 9. Radii in AC $GeCH_3$ PBE-optimized nanotubes. Computed average atomic radii with standard deviation for the AC $GeCH_3$ nanotubes at PBE level.

System	$R(H_1)$	$R(C_2)$	$R(Si_3)$	$R(O_4)$	R(Al5)	$R(O_6)$	$R(H_7)$
(9,9)	8.960±0.092	9.282±0.021	11.212±0.021	11.923±0.032	12.939±0.022	13.994±0.033	14.476±0.313
(10,10)	10.322±0.081	10.648±0.020	12.579±0.020	13.282±0.028	14.301±0.021	15.357±0.031	15.836±0.313
(11,11)	11.678±0.073	12.006±0.013	13.938±0.013	14.635±0.021	15.656±0.015	16.714±0.026	17.190±0.314
(12,12)	13.051±0.065	13.381±0.007	15.313±0.007	16.005±0.014	17.028±0.009	18.087±0.023	18.560±0.312
(13,13)	14.414±0.062	14.746±0.017	16.678±0.017	17.365±0.021	18.389±0.019	19.449±0.027	19.920±0.313
(14,14)	15.787±0.056	16.120±0.004	18.053±0.004	18.736±0.010	19.761±0.008	20.822±0.021	21.291±0.312
(15,15)	17.159±0.069	17.494±0.046	19.426±0.046	20.106±0.047	21.132±0.047	22.194±0.050	22.661±0.315
(16,16)	18.523±0.050	18.859±0.010	20.791±0.010	21.468±0.012	22.496±0.012	23.558±0.022	24.024±0.312
(17,17)	19.938±0.058	20.275±0.035	22.207±0.035	22.880±0.037	23.908±0.037	24.970±0.041	25.436±0.313
(18,18)	21.304±0.058	21.642±0.038	23.574±0.038	24.245±0.039	25.273±0.039	26.336±0.043	26.801±0.313
(19,19)	22.644±0.044	22.982±0.011	24.914±0.011	25.583±0.012	26.613±0.013	27.676±0.021	28.140±0.311
(20,20)	24.033±0.044	24.372±0.014	26.305±0.014	26.972±0.015	28.001±0.016	29.065±0.023	29.528±0.310

Supplementary Table 10. Radii in AC $GeCH_3$ PBE-D3-optimized nanotubes. Computed average atomic radii with standard deviation for the AC $GeCH_3$ nanotubes at PBE-D3 level.

Supplementary Note 10: Band-edges analysis of DFT-optimized structures

Vacuum-aligned Valence Band Edges (VBEs) for the AC and ZZ SiCH3 and GeCH3 nanotubes (Supplementary Figure 22) show that the reduced strain of the gibbsite O_4-Al_5-O_6 layer in the AC nanotubes leads to VBEs lower by as much as 0.3 eV with respect to ZZ values. Thus, for the same number of imogolite units *N*, bonding according to an AC geometry results in a substantially more favorable environment for the electrons of the nanotube, contributing to lower the energy of the entire system as shown in Supplementary Figure 22.

Electronic characterization of the AC and ZZ nanotubes in Supplementary Figure 22 reveals also that, whereas the $GeCH_3$ nanotube band gap (around 5 eV) is practically unaffected by AC or ZZ rolling, for $SiCH_3$ nanotubes AC rolling leads to 0.4 – 0.5 eV band-gap opening with respect to ZZ structures. For the specialist reader we also note that, given the expected underestimation of the PBE (PBE-D3) vacuum-aligned Conduction Band Edges (CBEs), the computed values close to the vacuum level indirectly points to the exact $SiCH_3$ CBE being possibly above the vacuum level.

The high-energy (vacuum aligned) valence bond edges (VBEs) of methylated ZZ nanotubes suggest strong energy drive for the nanotubes to act as hole scavenger for photo-catalysts grafted on the nanotube outer surface.[18-19] The high energy positioning of the VBE (in the -6.3/-6.0 eV range, Supplementary Figure 22) indicates that previous suggestions of strong hole-scavenging activity apply also to AC nanotubes.

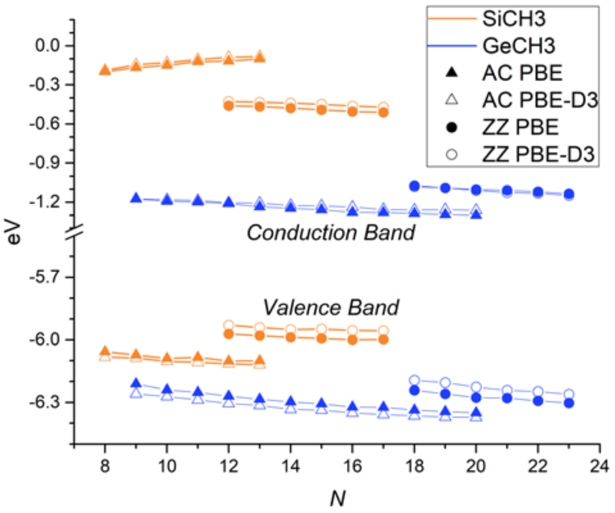

Supplementary Figure 22. Energies of the valence and conduction bands for AC and ZZ m-INTs. Vacuum-aligned Valence Band (VBE) and Conduction Band (CBE) edges for the AC (▲) and ZZ (●) $GeCH_3$ (blue) and $SiCH_3$ (orange) nanotubes at PBE (filled symbols) and PBE-D3 (hollow symbols) levels. The values displayed have been computed for the nanotubes with the DFT-optimized periods.

Supplementary Note 11: Electrostatic analysis of DFT optimized structures

Interpretation of electrophoresis experiments[16,17] and DFT calculation of hydroxylated imogolite nanotubes[15,18,19] agree on accumulation of (positive) negative charge on the (outer) inner surface of nanotube wall, leading to occurrence of a permanent dipole surface density (μ_σ). Such dipole density has been linked to the selective adsorption of ions inside and outside the NT cavity and the control of the bundling of nanotubes in powders, depending of the ionic strength of the initial suspension.[17] Recent DFT calculations of methylated ZZ $SiCH_3$ nanotubes indicate that the nanotube dipole density is qualitatively unaffected by methylation of the inner cavity, with preservation of the real-space separation between VBE and CBE inside and outside the cavity, respectively,[15,18,19] which prompts for investigation of the role of AC rolling and germanium substitution for the nanotube-wall dipole density.

By application of Gauss' theorem to two coaxial hollow cylinders of (opposite) charge (see Elliott et al.[19] for a full derivation of the electrostatic model) the nanotube dipole density μ_σ was obtained from the step in the electrostatic potential across the nanotube wall, that is, from the difference ($\Delta\bar{V} = \bar{V}_{\mathrm{in}} - \bar{V}_{\mathrm{out}}$) between the plateaus of the angularly and longitudinally-averaged electrostatic (ionic plus Hartree) potential inside ($\bar{V}_{\mathrm{in}}$) and outside ($\bar{V}_{\mathrm{out}}$) the nanotube cavity:

$$\mu_\sigma = -\frac{\Delta\bar{V}}{4\pi}\frac{\Delta R}{R_{\mathrm{in}}}\frac{1}{\ln\left(\frac{R_{\mathrm{in}}}{R_{\mathrm{out}}}\right)} = -\frac{\Delta\bar{V}}{4\pi}\frac{\Delta R}{R_{\mathrm{in}}}\frac{1}{\ln\left(\frac{R_{\mathrm{in}}}{R_{\mathrm{in}}+\Delta R}\right)} \#(5)$$

with the inner (R_{in}) and outer (R_{out}) radii defined as the onset of the vacuum-electrostatic plateaus inside and outside the nanotube, that is, the radii where the vacuum oscillations of $\bar{V}$ are smaller than an arbitrary (5×10^{-3} eV) threshold. $\Delta R = R_{\mathrm{out}} - R_{\mathrm{in}}$ is the electrostatic thickness of the INT wall. As the NTs are enclosed by vacuum in the directions perpendicular to their axis, no filtering or nanosmoothing[20] was applied.

For the sign convention used, positive μ_σ values indicate accumulation of negative (positive) charge-density at the inner (outer) surface of the NTs, with creation of electronegative (electropositive) environments inside (outside) the INT-cavity. Conversely, negative μ_σ values emerge from accumulation of positive (negative) charge-density at the inner (outer) surface of the nanotubes, with creation of electropositive (electronegative) environments inside (outside) the nanotube cavity.

We recall that, being based on the total electrostatic potential of the nanotube, this model by construction accounts for the combined role of the nanotube composition, atomic geometry (Supplementary Figure 11 to 20) and ensuing total (ion+electron) charge densities in determining an asymmetric total-charge distribution across the nanotube wall, thus generating an interface dipole-density and related potential step across the interface. For an extensive treatment on the computation of interface dipole densities from Density Functional Theory calculations accounting for atomic relaxation and total-charge redistribution effects (leading to changes in atom-decomposed charges, see for instance Supplementary Table 5 in the Supporting Information of Elliott et al.[19]), the interested reader is referred to Junquera et al.[20]

Electrostatic characterization of the nanotubes modelled (Supplementary Table 11-18) indicates that, owing to the different geometry and local bonding, AC rolling of $SiCH_3$ nanotubes reduces μ_σ by roughly 25% for the same number of imogolite units N (Supplementary Figure 23). In spite of this quantitative reduction in μ_σ, AC $SiCH_3$ nanotubes maintain the accumulation of the VBE (CBE) on the cavity (outer surface), typical of hydroxylated 'standard' imogolite nanotubes.[15,18,19] Conversely, we find that both AC and ZZ $GeCH_3$ INT present an inverted dipole-density: the positive (negative) end of μ_σ is now oriented towards the nanotube cavity (outer surface). Also at odds with the $SiCH_3$ case, AC $GeCH_3$ nanotubes are computed to have roughly 25% larger $|\mu_\sigma|$ with respect to ZZ ones, for the same number of imogolite units N. The inversion of μ_σ is found to be accompanied by inversion in the real-space localization of the VBE and CBE (Supplementary Figure 23). These differences further illustrate the potential of

functionalized INTs for controlling the electrostatics inside nanotubes' cavity, with potential interest for photo-catalytic applications and tuning the energy of confined molecular species.

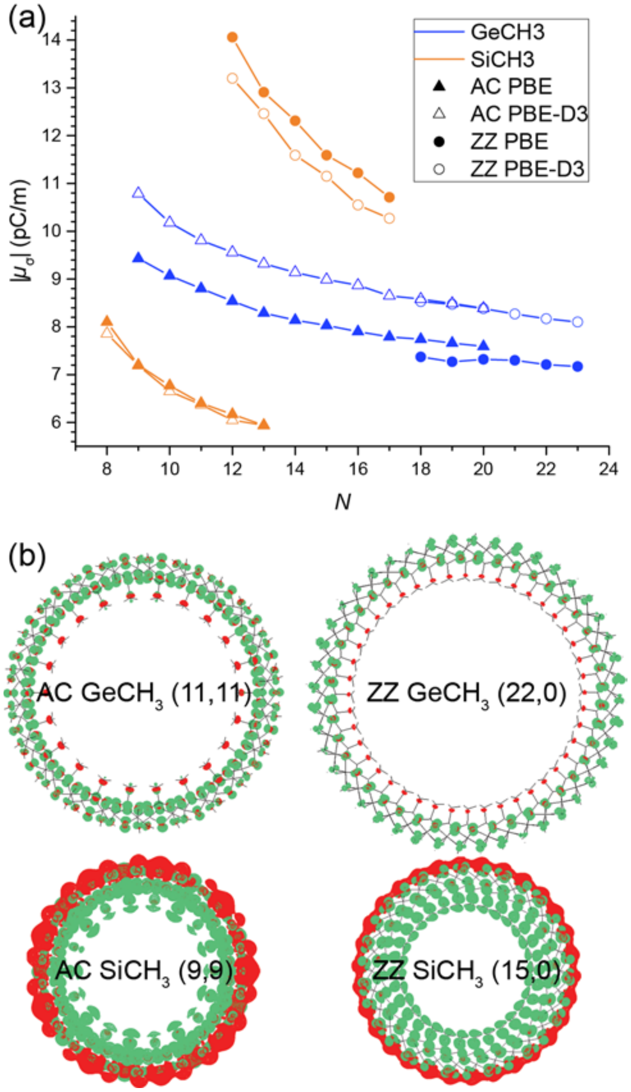

Supplementary Figure 23. Separation of charges in methylated imogolite nanotubes. (a) Computed nanotube wall dipole-density in absolute value ($|\mu_\sigma|$) for the AC (▲) and ZZ (●) $GeCH_3$ (blue) and $SiCH_3$ (orange) nanotubes at PBE (filled symbols) and PBE-D3 (hollow symbols) level (with optimized periods). (b) Front view of the real space separation between VBE (green) and CBE (red) for the PBE lowest-energy AC and ZZ nanotubes.

System	R_{in} (Å)	R_{out} (Å)	ΔR (Å)	$V(R_{in})$ (eV)	$V(R_{out})$ (eV)	ΔV (eV)	μ_σ (pC/m)
(12,0)	3.87	13.71	9.84	2.21	1.42	0.79	14.06
(13,0)	4.75	14.59	9.84	2.32	1.53	0.79	12.91
(14,0)	5.45	15.29	9.84	2.43	1.64	0.79	12.31
(15,0)	6.33	16.17	9.84	2.53	1.74	0.79	11.59
(16,0)	7.03	16.88	9.85	2.64	1.85	0.79	11.22
(17,0)	7.91	17.58	9.67	2.74	1.95	0.79	10.71

Supplementary Table 11. Computed electrostatic parameters and wall dipole-density (μ_σ) for ZZ $SiCH_3$ nanotubes at PBE level.

System	R_{in} (Å)	R_{out} (Å)	ΔR (Å)	$V(R_{in})$ (eV)	$V(R_{out})$ (eV)	ΔV (eV)	μ_σ (pC/m)
(12,0)	3.87	13.71	9.84	2.18	1.44	0.74	13.20
(13,0)	4.57	14.41	9.84	2.30	1.55	0.75	12.46
(14,0)	5.45	15.29	9.84	2.41	1.66	0.75	11.59
(15,0)	6.15	16.00	9.85	2.52	1.77	0.75	11.15
(16,0)	7.03	16.88	9.85	2.62	1.88	0.74	10.55
(17,0)	7.73	17.58	9.85	2.73	1.98	0.75	10.27

Supplementary Table 12. Computed electrostatic parameters and wall dipole-density (μ_σ) for ZZ $SiCH_3$ nanotubes at PBE-D3 level.

System	R_{in} (Å)	R_{out} (Å)	ΔR (Å)	$V(R_{in})$ (eV)	$V(R_{out})$ (eV)	ΔV (eV)	μ_σ (pC/m)
(8,8)	5.24	15.17	9.93	1.83	1.31	0.52	8.10
(9,9)	6.68	16.43	9.75	1.97	1.47	0.50	7.20
(10,10)	7.94	17.88	9.94	2.12	1.63	0.49	6.77
(11,11)	9.39	19.14	9.75	2.27	1.77	0.50	6.40
(12,12)	10.65	20.58	9.93	2.42	1.93	0.49	6.17
(13,13)	12.10	22.03	9.93	2.56	2.07	0.49	5.94

Supplementary Table 13. Computed electrostatic parameters and wall dipole-density (μ_σ) for AC $SiCH_3$ nanotubes at PBE level.

System	R_{in} (Å)	R_{out} (Å)	ΔR (Å)	$V(R_{in})$ (eV)	$V(R_{out})$ (eV)	ΔV (eV)	μ_σ (pC/m)
(8,8)	5.24	15.17	9.93	1.82	1.32	0.50	7.86
(9,9)	6.50	16.43	9.93	1.97	1.47	0.50	7.19
(10,10)	7.94	17.69	9.75	2.12	1.63	0.49	6.65
(11,11)	9.21	19.14	9.93	2.27	1.78	0.49	6.37
(12,12)	10.65	20.40	9.75	2.41	1.93	0.48	6.05
(13,13)	11.92	21.85	9.93	2.57	2.08	0.49	5.94

Supplementary Table 14. Computed electrostatic parameters and wall dipole-density (μ_σ) for AC $SiCH_3$ nanotubes at PBE-D3 level.

System	R_{in} (Å)	R_{out} (Å)	ΔR (Å)	$V(R_{in})$ (eV)	$V(R_{out})$ (eV)	ΔV (eV)	μ_σ (pC/m)
(18,0)	8.68	18.58	9.90	0.32	0.88	-0.56	-7.37
(19,0)	9.38	19.27	9.89	0.37	0.93	-0.55	-7.27
(20,0)	10.24	20.14	9.90	0.41	0.99	-0.58	-7.32
(21,0)	11.11	21.01	9.90	0.46	1.05	-0.59	-7.30
(22,0)	11.81	21.70	9.89	0.51	1.10	-0.59	-7.21
(23,0)	12.67	22.57	9.90	0.56	1.16	-0.60	-7.17

Supplementary Table 15. Computed electrostatic parameters and wall dipole-density (μ_σ) for ZZ $GeCH_3$ nanotubes at PBE level.

System	R_{in} (Å)	R_{out} (Å)	ΔR (Å)	$V(R_{in})$ (eV)	$V(R_{out})$ (eV)	ΔV (eV)	μ_σ (pC/m)
(18,0)	8.51	18.40	9.89	0.25	0.89	-0.64	-8.53
(19,0)	9.38	19.27	9.89	0.30	0.95	-0.65	-8.47
(20,0)	10.07	19.97	9.90	0.35	1.01	-0.66	-8.38
(21,0)	10.94	20.83	9.89	0.40	1.06	-0.66	-8.27
(22,0)	11.63	21.53	9.90	0.45	1.12	-0.67	-8.17
(23,0)	12.50	22.40	9.90	0.51	1.18	-0.67	-8.10

Supplementary Table 16. Computed electrostatic parameters and wall dipole-density (μ_σ) for ZZ $GeCH_3$ nanotubes at PBE-D3 level.

System	R_{in} (Å)	R_{out} (Å)	ΔR (Å)	$V(R_{in})$ (eV)	$V(R_{out})$ (eV)	ΔV (eV)	μ_σ (pC/m)
(9,9)	6.60	16.67	10.07	0.12	0.77	-0.65	-9.43
(10,10)	7.99	18.06	10.07	0.20	0.87	-0.67	-9.07
(11,11)	9.38	19.44	10.06	0.29	0.96	-0.67	-8.80
(12,12)	10.76	20.66	9.90	0.37	1.06	-0.69	-8.54
(13,13)	12.33	22.22	9.89	0.47	1.16	-0.69	-8.29
(14,14)	13.72	23.61	9.89	0.57	1.26	-0.69	-8.14
(15,15)	15.10	24.83	9.73	0.66	1.36	-0.70	-8.03
(16,16)	16.49	26.39	9.90	0.77	1.47	-0.70	-7.90
(17.17)	17.88	27.60	9.72	0.87	1.57	-0.70	-7.79
(18,18)	19.27	28.99	9.72	0.97	1.68	-0.71	-7.74
(19,19)	20.49	30.38	9.89	1.08	1.79	-0.71	-7.66
(20,20)	21.88	31.77	9.89	1.19	1.90	-0.71	-7.59

Supplementary Table 17. Computed electrostatic parameters and wall dipole-density (μ_σ) for AC $GeCH_3$ nanotubes at PBE level.

System	R_{in} (Å)	R_{out} (Å)	ΔR (Å)	$V(R_{in})$ (eV)	$V(R_{out})$ (eV)	ΔV (eV)	μ_σ (pC/m)
(9,9)	6.60	16.67	10.07	4.74	0.79	-0.75	-10.79
(10,10)	7.99	17.88	9.89	0.13	0.88	-0.75	-10.18
(11,11)	9.38	19.27	9.89	0.22	0.98	-0.76	-9.81
(12,12)	10.76	20.66	9.90	0.31	1.08	-0.77	-9.56
(13,13)	12.15	22.05	9.90	0.41	1.18	-0.77	-9.32
(14,14)	13.54	23.44	9.90	0.51	1.29	-0.78	-9.14
(15,15)	14.93	24.83	9.90	0.61	1.39	-0.78	-8.99
(16,16)	16.32	26.22	9.90	0.72	1.50	-0.78	-8.87
(17.17)	17.71	27.60	9.89	0.83	1.61	-0.78	-8.65
(18,18)	19.10	28.99	9.89	0.94	1.72	-0.78	-8.58
(19,19)	20.49	30.21	9.72	1.04	1.83	-0.79	-8.49
(20,20)	21.88	31.60	9.72	1.16	1.94	-0.78	-8.39

Supplementary Table 18. Computed electrostatic parameters and wall dipole-density (μ_σ) for AC $GeCH_3$ nanotubes at PBE-D3 level.

Supplementary References

1. Bishop, J. L. *et al.* Spectral and Hydration Properties of Allophane and Imogolite. *Clays Clay Min.* **61,** 57–74 (2013).
2. Amara, M. S. *et al.* Hybrid, Tunable-Diameter, Metal Oxide Nanotubes for Trapping of Organic Molecules. *Chem. Mater.* **27,** 1488–1494 (2015).
3. Hill, J.-R. & Sauer, J. Molecular Mechanics Potential for Silica and Zeolite Catalysts Based on Ab Initio Calculations. 2. Aluminosilicates. *J Phys Chem* **99,** 9536–9550 (1995).
4. Teppen, B. J., Rasmussen, K., Bertsch, P. M., Miller, D. M. & Schäfer, L. Molecular Dynamics Modeling of Clay Minerals. 1. Gibbsite, Kaolinite, Pyrophyllite, and Beidellite. *J Phys Chem B* **101,** 1579–1587 (1997).
5. Ermoshin, V. A., Smirnov, K. S. & Bougeard, D. Ab Initio Generalized Valence Force Field for Zeolite Modelling 2. Aluminosilicates. *Chem Phys* **209,** 41–51 (1996).
6. Etchepare, J., Merian, M. & Smetankine, L. Vibrational Normal Modes of SiO_2. I. α and β Quartz. *J Chem Phys* **60,** 1873–1876 (1974).
7. Creton, B. Étude par dynamique moléculaire du comportement d'aluminosilicates tubulaires hydratés: structure et dynamique du système eau-imogolite. (Université des Sciences et Technologie de Lille-Lille I, 2006).
8. Sastre, G. & Corma, A. Predicting Structural Feasibility of Silica and Germania Zeolites. *J Phys Chem C* **114,** 1667–1673 (2010).
9. Cottrell, T. L. *The Strengths of Chemical Bonds*. **42,** (Academic Press, 1958).
10. Saalfeld, H. & Wedde, M. Refinement of the crystal structure of gibbsite, $Al(OH)_3$. *Z. Für Krist.* **139,** 129–135 (1974).
11. Balan, E., Lazzeri, M., Morin, G. & Mauri, F. First-principles Study of the OH-stretching Modes of Gibbsite. *Am Miner.* **91,** 115–119 (2006).
12. Bougeard, D., Smirnov, K. S. & Geidel, E. Vibrational Spectra and Structure of Kaolinite: A Computer Simulation Study. *J. Phys. Chem. B* **104,** 9210–9217 (2000).
13. Yucelen, G. I. *et al.* Shaping Single-Walled Metal Oxide Nanotubes from Precursors of Controlled Curvature. *Nano Lett.* **12,** 827–832 (2012).
14. Mitsuyama, R. *et al.* Chirality fingerprinting and geometrical determination of single-walled carbon nanotubes: Analysis of fine structure of X-ray diffraction pattern. *Carbon* **75,** 299–306 (2014).
15. Teobaldi, G., Beglitis, N. S., Fisher, A. J., Zerbetto, F. & Hofer, W. A. Hydroxyl vacancies in single-walled aluminosilicate and aluminogermanate nanotubes. *J. Phys. Condens. Matter* **21,** 195301 (2009).
16. Gustafsson, J. P. The Surface Chemistry of Imogolite. *Clays Clay Miner.* **49,** 73–80 (2001).
17. Paineau, E. *et al.* Effect of Ionic Strength on the Bundling of Metal Oxide Imogolite Nanotubes. *J. Phys. Chem. C* **121,** 21740–21749 (2017).
18. Poli, E. *et al.* The potential of imogolite nanotubes as (co-)photocatalysts: a linear-scaling density functional theory study. *J. Phys. Condens. Matter* **28,** 074003 (2016).
19. Elliott, J. D. *et al.* Chemically Selective Alternatives to Photoferroelectrics for Polarization-Enhanced Photocatalysis: The Untapped Potential of Hybrid Inorganic Nanotubes. *Adv. Sci.* **4,** 1600153 (2017).
20. Junquera, J., Cohen, M. H. & Rabe, K. M. Nanoscale smoothing and the analysis of interfacial charge and dipolar densities. *J. Phys. Condens. Matter* **19,** 213203 (2007).
